# General Identifiability Condition for Network Topology Monitoring with Network Tomography

**DOI:** 10.3390/s19194125

**Published:** 2019-09-24

**Authors:** Shengli Pan, Zongwang Zhang, Zhiyong Zhang, Deze Zeng, Rui Xu, Zhihong Rao

**Affiliations:** 1 Hubei Key Laboratory of Intelligent Geo-Information Processing, School of Computer Science, China University of Geosciences, Wuhan 430078, China; 2 Cyberspace Security Key Laboratory of Sichuan Province & Cyberspace Security Technology Laboratory of CETC, China Electronic Technology Cyber Security Co. LTD., Chengdu 610041, China

**Keywords:** network monitoring, network tomography, end-to-end measurement, topology identifiability

## Abstract

Accurate knowledge of network topology is vital for network monitoring and management. Network tomography can probe the underlying topologies of the intervening networks solely by sending and receiving packets between end hosts: the performance correlations of the end-to-end paths between each pair of end hosts can be mapped to the lengths of their shared paths, which could be further used to identify the interior nodes and links. However, such performance correlations are usually heavily affected by the time-varying cross-traffic, making it hard to keep the estimated lengths consistent during different measurement periods, i.e., once inconsistent measurements are collected, a biased inference of the network topology then will be yielded. In this paper, we prove conditions under which it is sufficient to identify the network topology accurately against the time-varying cross-traffic. Our insight is that even though the estimated length of the shared path between two paths might be “zoomed in or out” by the cross-traffic, the network topology can still be recovered faithfully as long as we obtain the relative lengths of the shared paths between any three paths accurately.

## 1. Introduction

Many network functions such as network monitoring and management [1,2], diagnosis, optimization, and network operation, rely on the accurate knowledge of network topologies. For networks that enable the response of Internet Control Message Protocol (ICMP), one can make use of the “Raspberry Pi” [3] as the network monitoring nodes to build an overlay network [4], whose nodes run network tools such as *Traceroute* to collect information of Internet Protocol (IP) addresses from the Time-To-Live (TTL) timeout responses and piece together the underlying network topology [5]. However, the practical deployments of these network tools are sometimes quite limited, since more and more network operators are likely to remove the support of ICMP from their networks due to security concerns. As a result, network topology tomography, which employs end-to-end measurements to identify network topology [6], emerges as an appealing supplementary method. Regarding that network tomography generally assumes no cooperation from network intermediate elements, it thus can be well deployed to networks without ICMP responses.

**Figure 1:**
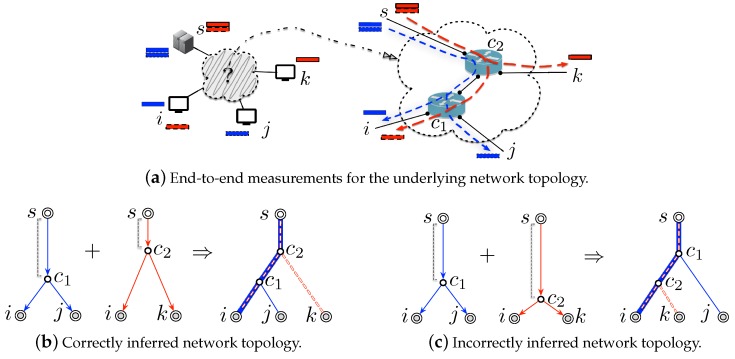
Illustration of network topology tomography.

By conducting end-to-end measurements on all pairwise paths, the correlation of performances (e.g., delay or loss rates [7,8,9,10]) between any two paths are measured and related to the length of their shared paths. Since such length indicates where two paths will split, how to accurately obtain them becomes the primary task in network topology tomography. For example, in Figure 1a, we send probes each composed of two “back-to-back” packets (colored in blue) from the source node *s* to the destination node *i* and *j*, for a pairwise delay measurement. Noticed that two “back-to-back” packets are closely spaced in the shared path connecting from *s* to $c_{1}$, their transmission delays then are supposed to be identical before they split on the branching node $c_{1}$, introducing corrections to the (end-to-end) path delays. To measure their delay correlation, one just needs to compute the delay covariance [11]. It is clear that longer the shared path is, larger the delay covariance will be. Consequently, one can efficiently determine the position of the branching nodes for network topology inference by comparing these delay covariances [12,13,14]. For example, when the computed delay covariance between the two paths destined at node *i* and *j* is larger than the one for those two paths destined at node *i* and *k*, the branching node $c_{1}$ then will be correctly placed under $c_{2}$, making an accurate inference of the network topology depicted in Figure 1b. For a more comprehensive review of network topology tomography, it is suggested to refer to the survey work of Zhang et al. [5].

However, as investigated in [15], such correlation measurements between two end-to-end paths are found to vary with the cross-traffic, which is also referred to background network traffic (or just background traffic) and is a common term in network tomography. It is used to denote the non-probing traffic that is passing in parallel to the probing traffic (i.e., injected by the end-to-end measurements [16]). For example, the delay covariances will be “zoom out” (i.e., be greater) when the cross-traffic gets a heavier load intensity. It might be reasonable for one to assume that the cross-traffic of a small-scaled network stays stable during the entire measurement period, in terms of that the number of all pairwise paths is small and the entire end-to-end measurements will not last long. However, if the network gets a large size, such measurement periods will take a long time, making it impractical for the cross-traffic to keep its load intensity unchanged [17]. Moreover, some differences inherently exist between the cross-traffic of the local networks, due to the network heterogeneity from their various network elements, configurations, and applications, etc. Once this is the case, inconsistent estimation of the shared path lengths will be generated, leading to inaccurate inference of the underlying topology. For instance, in Figure 1a, when the cross-traffic on the shared path between the pair of paths destined for node *i* and *k* becomes more intensive, the computed delay covariance will be comparably much larger. This will make $c_{2}$ wrongly placed under $c_{1}$, leading the network topology to be incorrectly inferred as Figure 1c. As you can see, due to the time-varying and heterogeneous cross-traffic [16], the practicality of current network topology tomography is significantly limited by its pairwise end-to-end measurements.

To this end, in this letter, we first explore the theoretical side of network topology tomography with triple-wise end-to-end measurements, which at a time probe three paths instead of a pair of paths. The key motivation behinds triple-wise end-to-end measurements is that links in the final inferred topology actually does not have any specific metric. They only represent the logical connections between nodes. Therefore, rather than using conventional pairwise end-to-end measurements to get the absolute length of the shared paths, triple-wise end-to-end measurements obtain their relative length. We theoretically show that once the sub-topology of any three paths can be accurately identified from triple-wise end-to-end measurements, the underlying network topology is identifiable. Our theoretical results indicate that one can deploy hybrid triple-wise measurement schemes (e.g., end-to-end probing alongside traceroute tracing) for different local parts of networks, as long as they can correctly obtain these sub-topologies.

## 2. Preliminaries

### 2.1. Topology Model and Terminology

Similar to most of works on network topology tomography, we also focus on the tree network inference since a general network topology can be well recovered by merging multiple tree networks [18,19]. We denote the network topology as a directed tree T≜(V,L), where V is the set of nodes interconnected by links in L. The source of probing packets is located at the root node s∈V, while the set of destinations or receivers are located at the leaf nodes from D. By convention of network tomography, the tree topology T is a logical one and any interior nodes of degree 2 will not be included [5]. An end-to-end path that consists of consecutive links is said to start at the root node *s* while ending at some leaf node. We use $p_{i}$ to refer to the end-to-end path ending at node i∈D, and collect the set of all the end-to-end paths in P. Moreover, the number of end-to-end paths in the intervening network T is required as ∥P∥≥3. Given $p_{i},p_{j}\in P$, let $p_{i}\wedge p_{j}$ be the shared path of $p_{i}$ and $p_{j}$. As previously stated, the length of this shared path is marked by $∥p_{i}\wedge p_{j}∥$, which measures how far the root node *s* stays from the branching node of $p_{i}$ and $p_{j}$.

We define the following two helper terms: one is ϕ(v) to denote the set of all end-to-end paths that traverse the node v∈V; the other is ϕ(ℓ) to collect the set of all end-to-end paths routing through the link ℓ∈L. As depicted in Figure 1, we get $\phi \left(c_{1}\right)≜\left\{p_{i},p_{j}\right\}$ and $\phi \left(\ell_{c_{2},k}\right)≜\left\{p_{k}\right\}$, where $\ell_{c_{2},k}$ marks the link connecting from node $c_{2}$ to node *k*. For the sake of proof, we also have $V_{\phi }≜\left\{\phi \left(v\right)|v\in V\right\}$ and $L_{\phi }≜\left\{\phi \left(\ell \right)|\ell \in L\right\}$, whose elements are ϕ(v) for ∀v∈V and ϕ(ℓ) for ∀ℓ∈L, respectively. E.g., in Figure 1, we have $V_{\phi }=\left\{\left\{p_{i},p_{j},p_{k}\right\},\left\{p_{i},p_{j},p_{k}\right\},\left\{p_{i},p_{j}\right\},\left\{p_{i}\right\},\left\{p_{j}\right\},\left\{p_{k}\right\}\right\}$ and $L_{\phi }=\left\{\left\{p_{i},p_{j},p_{k}\right\},\left\{p_{i},p_{j}\right\},\left\{p_{i}\right\},\left\{p_{j}\right\},\left\{p_{k}\right\}\right\}$.

### 2.2. Sub-Topology Composed of Three End-to-End Paths

**Figure 2:**
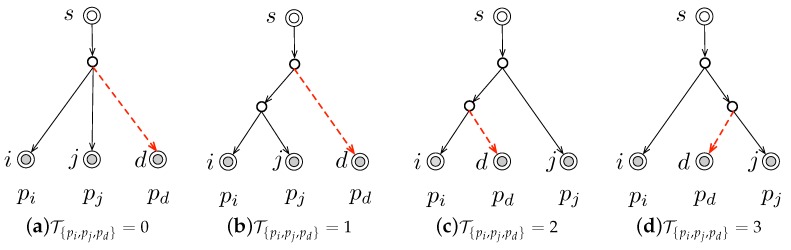
The four different kinds of three-path-based sub-topologies.

Given R⊂D, $T_{R}$ is the subtree of T composed of all the end-to-end paths corresponding to *R*. In this letter, we are especially interested in the subtrees composed of three end-to-end paths, i.e., ∥R∥=3. We enumerate and depict all the four different kinds of such subtrees in the following Figure 2.

The concrete structures of $T_{\left\{p_{i},p_{j},p_{d}\right\}}$ is illustrated in Figure 2. Specifically, $T_{\left\{p_{i},p_{j},p_{d}\right\}}=0$ denotes the sub-topology that all these three paths share the same branching node, indicating that the shared paths between each pair of end-to-end path are identical, i.e., $∥p_{i}\wedge p_{j}∥=∥p_{j}\wedge p_{d}∥=∥p_{i}\wedge p_{d}∥$; otherwise, we use $T_{\left\{p_{i},p_{j},p_{d}\right\}}=1$, $T_{\left\{p_{i},p_{j},p_{d}\right\}}=2$, and $T_{\left\{p_{i},p_{j},p_{d}\right\}}=3$, to indicate $∥p_{i}\wedge p_{j}∥>∥p_{j}\wedge p_{d}∥$, $∥p_{i}\wedge p_{d}∥>∥p_{j}\wedge p_{d}∥$, and $∥p_{j}\wedge p_{d}∥>∥p_{i}\wedge p_{j}∥$, respectively. For a subtree $T_{R}$, we denote ${\hat{T}}_{R}$ as its inferred network topology. More specifically, when ${\hat{T}}_{R}$ is obtained through inserting $p_{d}$ into (the true topology) $T_{R\backslash \{p_{d}\}}$, we especially mark it as ${\hat{T}}_{R\backslash \{p_{d}\}}^{p_{d}}$, where $R\backslash \{p_{d}\}$ denotes the set of paths by removing the path $p_{d}$ from the path set *R*. For the entire tree network T, it will be reconstructed initially with a subtree composed of three paths in Figure 2, and then by repetitively inserting the rest of end-to-end paths [12]. In the following section, we will theoretically demonstrate that as long as all such subtrees in Figure 2 are correctly obtained, T can be identified accurately.

## 3. Topology Identifiability with Three-Path-Based Sub-Topologies

In this section, we will prove the topology identifiability with mathematical induction. First, we theoretically demonstrate that the triple-wise end-to-end measurements will always generate the consistent sub-topologies for any three paths with Lemma 1 and Lemma 2, if these triple-wise end-to-end measurements are free of errors. Noticed that network topology recovery can be proceeded by recursively adding an end-to-end path to the current semi-recovered topology. Thus, secondly, we show that in Lemma 3 the topology inferred by adding a path to the current semi-recovered topology is also correct if the current semi-recovered topology is accurately inferred. Finally, we prove the topology identifiability in Theorem 1.

**Lemma** **1.** *Given T=(V,L), the node set V (resp. the link set L) and $V_{\phi }$ (resp. $L_{\phi }$) are bijective.*

**Proof of Lemma** **1.** We first prove that Lemma 1 holds true for the node set V and $V_{\phi }$. To this end, we need to prove that the map from V to $V_{\phi }$ is both injective and surjective. (a) Supposed that $\exists \;v_{1},v_{2}\in V$ such that $\phi \left(v_{1}\right)=\phi \left(v_{2}\right)$. This means that at least two paths are going to rejoin after they split, indicating that either $v_{1}$ or $v_{2}$ should be a joining node. However, a tree topology T gets no joining node in V. Thus, the above suppose does not hold, and we will have $\phi \left(v_{1}\right)\neq \phi \left(v_{2}\right)$ for any $v_{1},v_{2}\in V$, meaning that the map from V to $V_{\phi }$ is injective. (b) As we always can find a node v∈V for $\forall \;\phi \left(v\right)\in V_{\phi }$, the map from V to $V_{\phi }$ then is also surjective. According to (a) and (b), we get a bijection between V and $V_{\phi }$. Hence, Lemma 1 holds true for the node set V and $V_{\phi }$.

We now prove Lemma 1 for L and $L_{\phi }$. Recalling that a link can be represented by a tuple composed of its starting node and ending node, the ending nodes of any two links in the tree topology T then are different as neither of them could be a joining node. Consequently, ∀ℓ∈L, ∃ a node v∈V such that only the ending node of *ℓ* is *v*, indicating that L and V in T is one-to-one correspondence (i.e., bijective). Since the composition of two bijections is again a bijection, we conclude that the map from L to $L_{\phi }$ is bijective regarding the bijection between V and $V_{\phi }$ proved in the beginning. Hence, Lemma 1 holds true. □

**Lemma** **2.** *Given T=(V,L), $\forall \;p_{i}$, $p_{j}$, and $p_{d}$, the three-path-based sub-topology $T_{\left\{p_{i},p_{j},p_{d}\right\}}$ is unique.*

**Proof of Lemma** **2.** In the tree topology T, it is known that there is a unique branching node between every two end-to-end paths. Supposed $T_{\left\{p_{i},p_{j},p_{d}\right\}}$ is not unique. This means $T_{\left\{p_{i},p_{j},p_{d}\right\}}$ at least corresponds to two different three-path-based sub-topologies as illustrated in Figure 2. (**I**) If we have $T_{\left\{p_{i},p_{j},p_{d}\right\}}=0$ and $T_{\left\{p_{i},p_{j},p_{d}\right\}}=1$, it is obvious that $p_{i}$ and $p_{d}$ get two different branching nodes. Such conclusion also holds when we replace $T_{\left\{p_{i},p_{j},p_{d}\right\}}=1$ with $T_{\left\{p_{i},p_{j},p_{d}\right\}}=2$ or $T_{\left\{p_{i},p_{j},p_{d}\right\}}=3$; (**II**) If $T_{\left\{p_{i},p_{j},p_{d}\right\}}=1$ and $T_{\left\{p_{i},p_{j},p_{d}\right\}}=2$ (resp. $T_{\left\{p_{i},p_{j},p_{d}\right\}}=3$), $p_{d}$ and $p_{i}$ (resp. $p_{j}$) also get two different branching nodes; (**III**) If $T_{\left\{p_{i},p_{j},p_{d}\right\}}=2$ and $T_{\left\{p_{i},p_{j},p_{d}\right\}}=3$, $p_{d}$ and $p_{j}$ get two different branching nodes. Obviously, (**I**), (**II**), and (**III**) together enumerate all the six different cases for $T_{\left\{p_{i},p_{j},p_{d}\right\}}$ that gets two different values. However, all these cases are violated the tree structure of T, indicating that $T_{\left\{p_{i},p_{j},p_{d}\right\}}$ can only get a unique value. Hence, Lemma 2 holds true. □

Lemma 1 reveals that the end-to-end measurements upon paths are closely related to the network nodes and links while Lemma 2 indicates that the measurement results of the sub-topology of any three end-to-end path will stay consistent if such measurements are free of errors. In what follows, we will show that inferring the network topology by adding paths recursively is able to identify the true network topology.

**Lemma** **3.** *$\forall \;p_{i}$, $p_{j}$, and $p_{k}$∈P, if given $T_{\left\{p_{i},p_{j},p_{k}\right\}}$, then $\forall \;p_{d}\in P$, we have ${\hat{T}}_{P\backslash \{p_{d}\}}^{p_{d}}=T_{P}$.*

**Proof of Lemma** **3.** As illustrated in Figure 2, $p_{d}$ is inserted into $T_{P\backslash \{p_{d}\}}$ either at a node or on a link. Without loss of generality, we denote the insertion node as *x* (resp. the insertion link as *y*).

(**I**) Supposed that $p_{d}$ is inserted into $T_{P\backslash \{p_{d}\}}$ at the node *x*. We denote the true insertion node for ${\hat{T}}_{P\backslash \{p_{d}\}}^{p_{d}}=T_{P}$ by *v*. Supposed x≠v, i.e., ${\hat{T}}_{P\backslash \{p_{d}\}}^{p_{d}}\neq T_{P}$. According to Lemma 1, x≠v indicates that we have ϕ(x)≠ϕ(v) in $T_{P\backslash \{p_{d}\}}$. Then we must have $[\phi \left(x\right)\cup \phi \left(v\right)]\backslash \left[\phi (x)\cap \phi (v)\right]⊈\emptyset$. (**I**.1) If $\left[\phi (x)\cap \phi (v)\right]\subseteq \emptyset$, then we select $p_{i}\in \phi \left(v\right)$ and $p_{j}\in \phi \left(x\right)$. After inserting $p_{d}$ into $T_{P\backslash \{p_{d}\}}$ at node *x*, we obtain a three-path-based sub-topology ${\hat{T}}_{\left\{p_{i},p_{j},p_{d}\right\}}$. Because of x≠v, it is easy to find that ${\hat{T}}_{\left\{p_{i},p_{j},p_{d}\right\}}$ in ${\hat{T}}_{P\;\{p_{d}\}}^{p_{d}}$ is different from the given $T_{\left\{p_{i},p_{j},p_{d}\right\}}$ in the true $T_{P}$, where $p_{d}$ is inserted at node *v*. For example in Figure 2c,d, ${\hat{T}}_{\left\{p_{i},p_{j},p_{d}\right\}}$ could be 2 while $T_{\left\{p_{i},p_{j},p_{d}\right\}}$ could be 3; (**I**.2) If $\left[\phi (x)\cap \phi (v)\right]⊈\emptyset$, then we must have either ϕ(x)⊊ϕ(v), or vice versa. Without loss of generality, let ϕ(x)⊊ϕ(v), we are able to find $p_{i}\in \phi \left(x\right)$ and $p_{j}\in \left[\phi (v)\backslash \phi (x)\right]$. As depicted in Figure 2, we have ${\hat{T}}_{\left\{p_{i},p_{j},p_{d}\right\}}=2$ in ${\hat{T}}_{P\;\{p_{d}\}}^{p_{d}}$ while the given one in the true $T_{P}$ is $T_{\left\{p_{i},p_{j},p_{d}\right\}}=1$. Therefore, we can always find some ${\hat{T}}_{\left\{p_{i},p_{j},p_{d}\right\}}$ in ${\hat{T}}_{P\;\{p_{d}\}}^{p_{d}}$ inconsistent with the given $T_{\left\{p_{i},p_{j},p_{d}\right\}}$ in the true $T_{P}$ for case (**I**). However, this violates Lemma 2, which generally states that the sub-topology composed of $p_{i}$, $p_{j}$, and $p_{d}$ must be unique. Consequently, the assumption does not hold, and we must have x=v. Thus, for case (**I**), Lemma 3 holds true.

(**II**) Supposed that $p_{d}$ is inserted into $T_{P\backslash \{p_{d}\}}$ at the link *y*. According to $T_{P}$, the true insertion link for inserting $p_{d}$ into $T_{P\backslash \{p_{d}\}}^{p_{d}}$ is marked by *ℓ*. Supposed y≠ℓ. Let *e* be the ending node of link *ℓ* for ϕ(ℓ)=ϕ(e). Recalling that a link could be solely represented by its ending node (referring to the proof of Lemma 1), then dual to case (a), we also can find $p_{i}$, $p_{j}$, and $p_{d}$, whose sub-topology ${\hat{T}}_{\left\{p_{i},p_{j},p_{d}\right\}}$ in ${\hat{T}}_{P\backslash \{p_{d}\}}^{p_{d}}$ varies from the given sub-topology $T_{\left\{p_{i},p_{j},p_{d}\right\}}$ in the true $T_{P}$, violating the theoretical conclusion in Lemma 2. The assumption of (**II**) is not true and we must have y=ℓ. Lemma 3 also holds for case (**II**). Hence, Lemma 3 holds true. □

It is indicated by Lemma 3 that a correct network topology inference will be made as long as the previous inference is also correct. Since the network topology inference starts with a three-path-based sub-topology illustrated in Figure 2, then the correct inference of the entire network topology is recursively guaranteed by the accurate knowledge of all the three-path-based sub-topologies. This makes Lemma 3 play the key role of network topology identifiability in the following inductive proof of Theorem 1.

**Theorem** **1.** *$\forall \;p_{i}$, $p_{j}$, and $p_{k}$∈P, if given $T_{\left\{p_{i},p_{j},p_{k}\right\}}$, we have ${\hat{T}}_{P}=T_{P}$.*

**Proof of Theorem** **1.** We employ mathematical induction to prove Theorem 1. When ∥P∥=3, it is obvious that Theorem 1 holds true. It is also easy to verify that Theorem 1 also holds true when ∥P∥=4. Now, we suppose Theorem 1 holds true for ∥P∥=k, where we have k≥3. We prove it that Theorem 1 will still hold true for ∥P∥=k+1.

We denote $P=P^{\prime }\cup \left\{p_{d}\right\}$, where accordingly we have $∥P^{\prime }∥=k$ and ∥P∥=k+1. As Theorem 1 holds for $∥P^{\prime }∥=k$, we get ${\hat{T}}_{P^{\prime }}=T_{P^{\prime }}$. Then recalling that we have already been given $T_{\left\{p_{i},p_{j},p_{k}\right\}}$ for $\forall \;p_{i}$, $p_{j}$, and $p_{k}\in P$, there must be ${\hat{T}}_{P^{\prime }}^{p_{d}}={\hat{T}}_{P\backslash \{p_{d}\}}^{p_{d}}=T_{P}$ according to Lemma 3. Consequently, Theorem 1 also holds for ∥P∥=k+1. The mathematical induction is completed. Hence, Theorem 1 holds true. □

## 4. Discussions on the Design of Triple-Wise End-to-End Measurements

After conducting end-to-end measurements, shared path lengths between two end-to-end paths can be estimated with metrics such as delay covariances, loss rates, and so on. However, the heterogeneity from applications, routing policies/devices, and user population in each local network, etc., generally makes it hard for their cross-traffic to keep stable for consistent estimation. As investigated in [15], inconsistency of such estimations will result in a low inference accuracy if end-to-end measurements are conducted with the same probing scheme (e.g., “back-to-back” probing) throughout the entire network regardless the heterogeneity of the cross-traffic. Consequently, before deploying the triple-wise end-to-end measurements, one should first be able to sense the load conditions of the cross-traffic for each path. For example, a potential way to sense the load insensitivity of the cross-traffic could be to constantly monitor the delay variance for each path.

**Figure 3:**
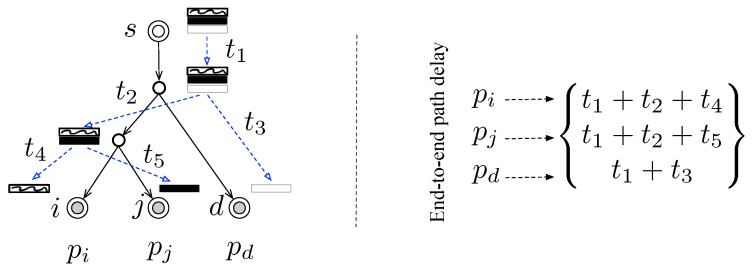
Triple-wise end-to-end measurements of path delay.

Once the intensity of the cross-traffic is obtained, the end-to-end measurement scheme could be selected appropriately according to [15]. Specifically, the triple-wise end-to-end measurement can be implemented with “sandwich” probing [7] for the light cross-traffic, “back-to-back” probing for the moderate cross-traffic; or “loss” measurements when in a lossy network such as the wireless sensor network. For example, when the intervening paths bare a moderate-load cross-traffic, we can follow the line of “back-to-back” probing to design the triple-wise end-to-end delay measurement scheme as depicted in Figure 3. Three closely spaced packets are sent from the source node *s* to each of the destination node *i*, *j*, and *d*. After collecting a sufficient number of end-to-end path delay samples for path $p_{i}$, $p_{j}$, and $p_{d}$, we are able to pairwise calculate the delay covariances for the estimation of $T_{\left\{p_{i},p_{j},p_{d}\right\}}$ effectively.

As implied by Theorem 1, if fortunately some parts of the intervening network support ICMP query, such triple-wise end-to-end measurement could even be carried out in conjunction with traceroute [20] in terms of Lemma 2. Moreover, our Theorem 1 also indicates that probing four (e.g., quadruple-wise) or more end-to-end paths at a time is feasible. Although so, the biggest challenge of the triple-wise end-to-end measurements might lie on the probing complexity [16], which could be as complex as O(3), i.e., the total number of combinations of selecting three paths from P is as large as ∥P∥3=∥P∥·(∥P∥−1)·(∥P∥−2)/6. For the quadruple-wise end-to-end measurements, its probing complexity could be O(4), which will give rise to a serious question of practicality when conducted in large-scale networks. Such a high probing complexity will manifest itself when conducting triple-wise end-to-end measurements in a large-scale network. It is suggested to refer to some heuristics, as in [13] the depth-first search, for a potential reduction in the probing complexity. Moreover, one can also follow the divide-and-rule tactics to solve the high probing complexity of the large-scale network. Specifically, they might first partition a large-scale network into multiple proper-scale sub-networks, and then conduct end-to-end measurements upon them. Through merging the topologies of these sub-networks [19,21], the original topology of the large-scale network will be reconstructed eventually. Moreover, errors exist during the triple-wise end-to-end measurements might also need to be carefully modeled regarding of practicability.

## 5. Conclusions

Network topology tomography is a good complementary approach when directly monitoring the network topology is not an option. However, most of current methods in network topology tomography are built upon the pairwise end-to-end measurements, such as the “back-to-back” probing, and often suffer an inconsistent inference of the network topology due to the heterogeneity of cross-traffic. In this paper, we prove that the underlying network topology can be accurately inferred if the sub-topology of any three end-to-end paths is given. Our proposed topology identifiability theory suggests to focusing on the accurate measurement of the three-path-based sub-topology, but regardless of the metrics used by different measurement techniques to identify such sub-topology. It shows that the triple-wise end-to-end measurements throughout the intervening network could be hybrid. We also present that according to the load conditions of local networks, one might achieve this by appropriately designing the triple-wise end-to-end measurement schemes, or even conduct traceroute to directly trace their sub-topology. For future work, a practical implementation of network topology probing and inference based on our theory, where it is able to be intelligently adaptive to various cross-traffic, will be included by the on-going research efforts with unsupervised methods [22] for network topology tomography.

## References

[1] Raposo D., Rodrigues A., Sinche S., Sa Silva J., Boavida F. (2018). Industrial IoT Monitoring: Technologies and Architecture Proposal. Sensors.

[2] Zhao N., Liang Y., Pei Y. (2018). Dynamic contract incentive mechanism for cooperative wireless networks. IEEE Trans. Veh. Technol..

[3] Hogg S. Raspberry Pi as a Network Monitoring Node. https://www.networkworld.com/article/2225683/cisco-subnet-raspberry-pi-as-a-network-monitoring-node.html.

[4] Chen J., Yang J. (2019). Maximizing Coverage Quality with Budget Constrained in Mobile Crowd-Sensing Network for Environmental Monitoring Applications. Sensors.

[5] Zhang X., Phillips C. (2011). A survey on selective routing topology inference through active probing. IEEE Commun. Surv. Tutor..

[6] Haddadi H., Rio M., Iannaccone G., Moore A., Mortier R. (2008). Network topologies: Inference, modeling, and generation. IEEE Commun. Surv. Tutor..

[7] Coates M., Castro R., Nowak R., Gadhiok M., King R., Tsang Y. Maximum likelihood network topology identification from edge-based unicast measurements. Proceedings of the 2002 ACM SIGMETRICS international conference on Measurement and Modeling of Computer Systems.

[8] Zhang R., Li Y., Li X. (2014). Topology Inference with Network Tomography Based on t-test. IEEE Commun. Lett..

[9] Nguyen H., Zheng R. (2013). A Binary Independent Component Analysis Approach to Tree Topology Inference. IEEE Trans. Signal Process..

[10] Bowden R., Veitch D. (2018). Finding the Right Tree: Topology Inference Despite Spatial Dependences. IEEE Trans. Inf. Theory.

[11] Duffield N.G., Presti F.L. (2004). Network tomography from measured end-to-end delay covariance. IEEE/ACM Trans. Netw..

[12] Ni J., Tatikonda S. (2011). Network tomography based on additive metrics. IEEE Trans. Inf. Theory.

[13] Erikson B., Dasarathy G., Barford P., Nowak R.D. (2012). Efficient Network Tomography for Internet Topology Discovery. IEEE/ACM Trans. Netw..

[14] Qin P., Dai B., Xu G., Wu K., Huang B. (2016). Taking a free ride for routing topology inference in peer-to-peer networks. Peer-to-Peer Netw. Appl..

[15] Shih M.F., Hero A.O. (2007). Hierarchical inference of unicast network topologies based on end-to-end measurements. IEEE Trans. Signal Process..

[16] Jaggard A., Kopparty S., Ramachandran V., Wright R.N. The design space of probing algorithms for network-performance measurement. Proceedings of the ACM SIGMETRICS/International Conference on Measurement and Modeling of Computer Systems.

[17] Khan A.A., Ghani S., Siddiqui S. (2018). A Preemptive Priority-Based Data Fragmentation Scheme for Heterogeneous Traffic in Wireless Sensor Networks. Sensors.

[18] Rabbat M.G. (2003). Multiple-Source Network Tomography. Ph.D. Thesis.

[19] Sattari P., Kurant M., Anandkumar A., Markopoulou A., Rabbat M.G. (2014). Active Learning of Multiple Source Multiple Destination Topologies. IEEE Trans. Signal Process..

[20] Di Pietro A., Ficara D., Giordano S., Oppedisano F., Procissi G., Vitucci F. Merging spanning trees in tomographic network topology discovery. Proceedings of the 2009 IEEE International Conference on Communications.

[21] Ettehad M., Duffield N., Berkolaiko G. (2019). Optimizing Consistent Merging and Pruning of Subgraphs in Network Tomography. arXiv.

[22] Qian F., Yin M., Liu X.Y., Wang Y.J., Lu C., Hu G.M. (2018). Unsupervised seismic facies analysis via deep convolutional autoencoders. Geophysics.

